# Three Measures of Forest Fire Smoke Exposure and Their Associations with Respiratory and Cardiovascular Health Outcomes in a Population-Based Cohort

**DOI:** 10.1289/ehp.1002288

**Published:** 2011-06-09

**Authors:** Sarah B. Henderson, Michael Brauer, Ying C. MacNab, Susan M. Kennedy

**Affiliations:** 1 School of Environmental Health, and; 2 School of Population and Public Health, University of British Columbia, Vancouver, British Columbia, Canada

**Keywords:** biomass smoke, cohort study, exposure assessment, particulate matter, population-based

## Abstract

Background: During the summer of 2003 numerous fires burned in British Columbia, Canada.

Objectives: We examined the associations between respiratory and cardiovascular physician visits and hospital admissions, and three measures of smoke exposure over a 92-day study period (1 July to 30 September 2003).

Methods: A population-based cohort of 281,711 residents was identified from administrative data. Spatially specific daily exposure estimates were assigned to each subject based on total measurements of particulate matter (PM) ≤ 10 μm in aerodynamic diameter (PM_10_) from six regulatory tapered element oscillating microbalance (TEOM) air quality monitors, smoke-related PM_10_ from a CALPUFF dispersion model run for the study, and a SMOKE exposure metric for plumes visible in satellite images. Logistic regression with repeated measures was used to estimate associations with each outcome.

Results: The mean (± SD) exposure based on TEOM-measured PM_10_ was 29 ± 31 μg/m^3^, with an interquartile range of 14–31 μg/m^3^. Correlations between the TEOM, smoke, and CALPUFF metrics were moderate (0.37–0.76). Odds ratios (ORs) for a 30-μg/m^3^ increase in TEOM-based PM_10_ were 1.05 [95% confidence interval (CI), 1.03–1.06] for all respiratory physician visits, 1.16 (95% CI, 1.09–1.23) for asthma-specific visits, and 1.15 (95% CI, 1.00–1.29) for respiratory hospital admissions. Associations with cardiovascular outcomes were largely null.

Conclusions: Overall we found that increases in TEOM-measured PM_10_ were associated with increased odds of respiratory physician visits and hospital admissions, but not with cardiovascular health outcomes. Results indicating effects of fire smoke on respiratory outcomes are consistent with previous studies, as are the null results for cardiovascular outcomes. Some agreement between TEOM and the other metrics suggests that exposure assessment tools that are independent of air quality monitoring may be useful with further refinement.

Forest fire smoke is a globally important source of particulate matter (PM) pollution (Andreae and Merlet 2001), but its public health effects are challenging to assess because smoke exposures are typically sporadic, short-lived, and rare in areas of high population density. Most epidemiologic studies have used PM data from routine air quality monitoring to estimate population exposure, consistently detecting some association between forest fire smoke and respiratory health (Duclos et al. 1990; Emmanuel 2000; Johnston et al. 2002, 2007; Kunii et al. 2002; Künzli et al. 2006; Lipsett et al. 1997; Moore et al. 2006; Mott et al. 2002, 2005; Tham et al. 2009; Wiwanitkit 2008). Less consistency has been observed for cardiovascular outcomes. Although we have strong evidence that acute exposure to wood smoke triggers a systemic inflammatory response (Barregard et al. 2006; Swiston et al. 2008; Tan et al. 2000) and that chronic exposure to urban fine PM increases cardiovascular morbidity and mortality (Dockery 2001; Pope et al. 2004), studies of acute exposure to forest fire smoke continue to report null findings for cardiovascular outcomes (Delfino et al. 2009; Hanigan et al. 2008; Johnston et al. 2007; Moore et al. 2006; Naeher et al. 2007).

Fire smoke epidemiology can be advanced by developing new, testable methods for estimating population exposure in places without air quality monitoring. For example, vast areas of South America, Africa, and Asia are cyclically affected by fire smoke, but valuable evidence from these regions is limited. Indeed, work related to the extreme smoke haze of 1997/1998 in Southeast Asia (Heil and Goldammer 2001) is crucial to the current body of literature, providing the only significant association between smoke and mortality yet reported (Sastry 2002). New methods could also help to improve smoke exposure assessment in areas where air quality is monitored, thereby reducing bias due to misclassification. Our primary objective was to design and implement a rigorous epidemiologic study on the population health effects of forest fire smoke exposure as estimated by surface measurements of PM mass concentrations. Our second objective was to evaluate whether exposure assessment methods based on plume dispersion modeling and remote sensing were similar to those based upon surface measurements of PM.

More than 2,600 km^2^ of forest were consumed in the southern interior of British Columbia, Canada, during the unprecedented forest fire season of 2003. A total of 343 homes were destroyed (Filmon 2004), and approximately 640,000 residents were potentially exposed to smoke pollution. In an unrelated study, Moore et al. (2006) used an ecologic design to associate forest fire smoke with increased respiratory physician visits in two large communities. We therefore felt confident about trying to generate better evidence using more rigorous epidemiologic methods in a larger study area. We used administrative health data to identify a population-based cohort of individuals who regularly used the health care system and who had geocodable residential addresses on file for the summer of 2003. We began by quantifying the association between 24-hr average total measurements of PM ≤ 10 μm in aerodynamic diameter (PM_10_) and physician visits/hospital admissions for respiratory and cardiovascular diseases. We derived the PM data for each cohort member from the nearest of six tapered element oscillating microbalance (TEOM) monitors used for routine air quality monitoring in the study area. We then compared associations of the health outcomes for the TEOM-based PM_10_ exposure estimates with associations for 24-hr average PM_10_ concentrations estimated using a smoke dispersion model (Henderson et al. 2008) and associations for a binary indicator of smoke plume coverage based on satellite imagery.

## Methods

*Study area and period.* The study covers the southeastern corner of the province of British Columbia in Canada. This area is bounded by the Alberta border to the northeast, the U.S. border to the south, and various geographic features (rivers, mountains, etc.) to the northwest. The study period was 92 days between 1 July and 30 September 2003. This period was chosen because it reflects the forest fire season in British Columbia.

*Administrative health data.* Most residents of British Columbia have a personal health number (PHN) and are registered for public health care through the provincial Medical Services Plan. A PHN is first issued at birth or upon immigration and is retired when a person dies. The Ministry of Health maintains databases of all records generated by every PHN, and researchers are able to apply for access to de-identified data. Approval is granted by the ministry on a case-by-case basis after the public health merits of the application have been assessed.

The only spatial attribute held in the health care billings file is a postal code, but a historical record of residential addresses is retained in the ministry client registry. Health care users are asked to confirm their last known address every time they use their PHN, and changes are flagged for amendment in this master file. When people change residences, their addresses are incorrect in the client registry until they contact the Ministry of Health directly to update their records, they update their address when they next use their PHN, or their employers update their address when paying annual fees for premium insurance. When none of these actions are taken, the addresses in the client registry will remain indefinitely incorrect. Because exposure assignment for this study is based on residential address, we endeavored to minimize misclassification by restricting the cohort to people with regular billings from the same postal code (thereby maximizing our confidence that the correctness of the postal code had been confirmed by recent contact with the health care system).

*Cohort identification and geolocation.* An individual was eligible for inclusion in the cohort only if the postal code associated with the last record generated by their PHN in the year before the study period (1 July 2002 through 30 June 2003) matched the postal code associated with the first record generated during the study period (1 July through 31 September 2003) or in the next year (1 October 2003 through 31 September 2004). All babies born during the study period were eligible. Once eligibility was established, an individual was included in the cohort only if that individual had a reliably geocodable (i.e., an accuracy ranking of 2 on Google’s geocoding utility) residential address in the ministry client registry during the summer of 2003. Many people in the study area live in rural and semirural areas where one six-digit postal code can cover thousands of square kilometers, so we assigned exposure based on addresses rather than postal codes to further reduce exposure misclassification.

**Figure 1:**
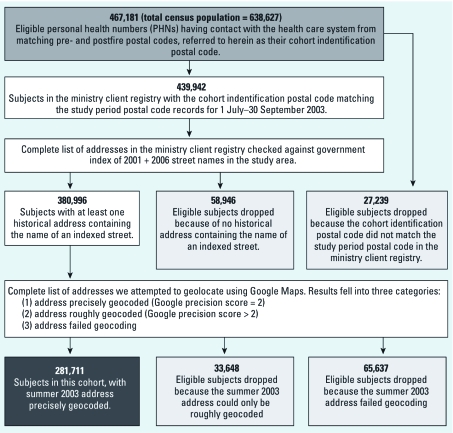
Outline of the cohort selection and geolocation process.

To protect the personal privacy of cohort members, their spatial information was never linked directly to their health information. Instead, the ministry provided a list of all street addresses in the client registry, and we used the batch geocoding capability of Google Maps to precisely locate each address. Any individual who had an address that could not be precisely geolocated was dropped from the cohort. Figure 1 outlines the details of this process. We then used ArcGIS (version 9.1; ESRI, Redlands, CA, USA) to impose a 1-km^2^ grid over the 500- × 650-km rectangle encompassing the study area (resulting in a total of 325,000 grid cells). Each of the geolocated addresses was assigned to the grid cell that contained it (referred to herein as its “exposure cell”), with 2,538 of the 325,000 grid cells containing cohort addresses [see Supplemental Material, Figure 1 (http://dx.doi.org/10.1289/ehp.1002288)]. Every address within each exposure cell was assigned the same daily exposure values as estimated at the central coordinates of that cell. This grid size was chosen to correspond to the modeling domain in Henderson et al. (2008). The addresses and their corresponding exposure data, including three daily measures of smoke exposure (described below) and daily mean temperature, were returned to the ministry. Ministry staff used the address field to link health data with exposure data for all cohort members and returned the file to us after redacting all spatial information and personal identifiers.

*Event definition.* Each record in the Medical Services Plan physician visit file includes a PHN, date of service, and one *International Classification of Diseases, 9th Revision* (ICD-9) (World Health Organization 1975), code describing the reason for the visit. We discarded all records for billings from physicians outside of the study area. A respiratory physician visit was defined as any billing with an ICD-9 code in the 460–519 range, and a cardiovascular physician visit was defined as any billing in the 390–459 range. For any individual having more than one event within 7 days, only the first event was included to avoid overcounting a single episode (e.g., a follow-up visit). A binary status indicator for each of the health outcomes assessed (1 = event, 0 = no event) was assigned to every cohort member or each of the 92 days in the study period. Hospital admissions data (also including the PHN, date, and ICD-9 codes for each admission) are recorded in a separate file and were treated in exactly the same way as the physician billing data, with events classified based on the primary ICD-9 code for each admission.

*Exposure metrics.* TEOM. Provincial and federal environmental authorities maintain PM_10_ TEOM instruments in the towns of Kelowna, Kamloops, Vernon, Creston, Revelstoke, and Golden [see Supplemental Material, Figure 1 (http://dx.doi.org/10.1289/ehp.1002288)]. We received hourly measurements from the British Columbia Ministry of Environment and used them to calculate midnight-to-midnight 24-hr average concentrations at each site. The average for any date with < 18 hourly measurements was set to missing. All subjects within each exposure cell were assigned daily concentrations from the TEOM nearest to the center of that cell. Calculations were made using the “path distance” function of ArcGIS, which accounts for the vertical and horizontal aspects of the topography between points. For example, an exposure cell in a valley would be considered nearer to a TEOM in the same valley than to a TEOM on the other side of a mountain, even if the linear distance to the latter was shorter.

CALPUFF. We previously used fire detection data from MODIS (Moderate Resolution Imaging Spectroradiometer) instruments to estimate PM emissions in a CALMET/CALPUFF smoke dispersion model (originally developed by the California Air Resources Board) (Henderson et al. 2008). Simulated hourly concentrations were averaged to estimate 24-hr PM_10_ concentrations at the center of each exposure cell for all subjects within that cell. The CALPUFF model estimates reflect smoke-related PM_10_ only, whereas the TEOM estimates reflect PM_10_ from all sources.

SMOKE. The U.S. National Oceanic and Atmospheric Administration Hazard Mapping System (http://www.firedetect.noaa.gov) provides information on the shape of smoke plumes based on expert review of satellite imagery, as described elsewhere (Ruminski et al. 2006). Data indicate the shape of smoke plumes at the time when the satellites are passing over, which happens multiple times daily. We combined all information for each 24-hr period to derive a single shape representing all areas covered by SMOKE during any part of the day. For days on which the center of an exposure cell fell within the SMOKE area, all subjects within that cell received a value of 1, and otherwise received a value of 0. Although these data provide information on the spatial extent of smoke impacts, they contain no information about surface PM_10_ concentrations.

For each health outcome assessed, we estimated odds ratios (ORs) associated with an increase of 30 μg/m^3^ (1 SD of the population exposure) in total PM_10_ based on TEOM measurements, an increase of 60 μg/m^3^ (1 SD of the population exposure) in smoke-related PM_10_ based on CALPUFF model estimates, and the presence versus absence of fire smoke based on the SMOKE metric. We evaluated SD increases instead of interquartile range increases in PM_10_ because the smoke-related PM_10_ estimates from CALPUFF are dominated by near-zero values, in contrast with estimates of total PM_10_ based on TEOM measurements. In addition, we hypothesized that a 1-SD change in PM_10_ based on either measure would result in SMOKE detection via satellite imagery. To facilitate comparisons with other studies, we also estimated ORs associated with a 10-μg/m^3^ increase in total PM_10_ based on TEOM measurements.

*Potential effect modifiers.* In addition to the time-varying exposures and outcomes described above, we evaluated potential effect modification by age at the beginning of the study period, sex, and socioeconomic status. The latter was estimated using the income quintile of the neighborhood in which the cohort member lived. These census regions typically contain 400–700 people, and quintiles are based on values from across Canada. In addition, we evaluated potential effect modification according to the number of physician visits in the year before the study period (0, 1–2, 3–5, ≥ 6 visits for respiratory ICD-9 codes, and 0, 1–2, 3–5, ≥ 6 visits for cardiovascular ICD-9 codes, 1 July 2002 through 30 June 2003) as a potential indicator of preexisting sensitivity to fire smoke. No information on other variables such as smoking or specific comorbidities was available from the administrative databases.

*Statistical analyses.* Logistic regression with repeated measures was used to estimate the independent fixed effects of a 30-μg/m^3^ increase in total PM_10_ (TEOM), a 60-μg/m^3^ increase in smoke-related PM_10_ (CALPUFF), and the presence or absence of exposure to a fire smoke plume (SMOKE) on all respiratory physician visits, all cardiovascular physician visits, all respiratory hospital admissions, and all cardiovascular hospital admissions. More specific analyses were also conducted for physician visits for asthma (ICD-9 code 493), acute upper respiratory infections (ICD-9 codes 465 and 466), and nonhypertensive cardiovascular diagnoses (ICD-9 codes 410–459). Stratified analyses were used to assess effect modification by age, sex, socioeconomic status, and possible preexisting sensitivity (based on numbers of respiratory or cardiovascular physician visits in the prior year). Coefficients were calculated with generalized estimating equations using the GENMOD procedure in SAS (version 9.1; SAS Institute Inc., Cary, NC, USA) and assuming an exchangeable correlation structure (where the correlation between all pairs of repeated measures within-subject is uniform and nonzero). Effect estimates were adjusted with linear terms for mean same-day temperature, day of week (0 = weekend/holiday, 1 = Monday, 2 = Tuesday . . . 5 = Friday), and week of study (1–13).

Because models including the entire cohort took approximately 48 hr to run (quad-core Intel Xeon 3500 computer; Dell, Toronto, ON, Canada), we conducted a preliminary evaluation of lag structures (between 0 and 7 days) based on data from potentially sensitive individuals with three or more respiratory/cardiovascular physician visits in the year before the study period. Lag 0 (i.e., exposure on the same day) was most consistently associated with increased risk across health effects and was chosen for all models to facilitate comparison across metrics. Preliminary results for other lags (TEOM metric) are reported in the Supplemental Material, Figures 2–6 (http://dx.doi.org/10.1289/ehp.1002288).

**Figure 2:**
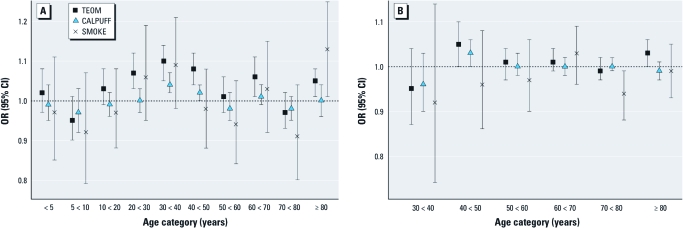
Models for respiratory physician visits (*A*) and cardiovascular visits (*B*) stratified on age. People < 30 years of age are omitted from the cardiovascular plot because of low event frequency (see Table 1). All results are for lag 0, and all models are adjusted for same-day mean temperature, day of week, and week of study. The ORs reflect a 30‑μg/m^3^ increase in total (TEOM-measured) PM_10_, a 60‑μg/m^3^ increase in smoke-related (CALPUFF-estimated) PM_10_, and the comparison of SMOKE and no SMOKE.

## Results

*Events summary.* Figure 1 summarizes the methods used to define the cohort and to identify the 281,711 subjects included in this study. Table 1 summarizes characteristics of the study population, which was 44.6% male and had a median (interquartile range) age of 45 (23–62) years. Cohort members were disproportionately split among neighborhood income quintiles, with approximately 23% in the lowest category and approximately 18% in the other categories (Table 1). Table 1 also summarizes the overall and stratified outcome frequencies. The mean (± SD) number of daily physician visits was 378 ± 174 and 500 ± 291 for respiratory and cardiovascular ICD-9 codes, respectively. Of the 34,771 respiratory physician visits, 5,496 (16%) were coded as “asthma” (ICD-9 code 493). The mean number of daily hospital admissions was 6.1 ± 3.6 and 13.1 ± 4.3, respectively. We observed a decreasing gradient in event frequency between Monday and Friday for all outcomes (data not shown), with physician visits being considerably lower on weekends and holidays (resulting in wide SDs).

**Table 1:** Percentages of cohort characteristics and health outcomes during the 92-day study period, by categories of the ordinal non-time-varying covariates.

Respiratory					Cardiovascular		
Variable	Whole cohort	All physician visits	Asthma physician visits	All hospital admissions	All physician visits	All hospital admissions
*n*		281,711		34,711		5,496		557		46,027		1,208
Sex												
Male		44.6		41.5		40.5		54.2		46.3		55.1
Female		55.4		58.5		59.5		45.8		53.7		44.9
Age (years)												
0 < 5		4.3		8.0		6.1		9.2		0.1		0.2
5 < 10		5.0		6.6		6.8		4.7		0.0		0.1
10 < 20		12.3		13.2		13.6		4.5		0.3		0.1
20 < 30		9.5		10.0		10.5		3.4		0.9		0.6
30 < 40		11.5		10.9		13.4		3.8		2.2		1.3
40 < 50		15.4		12.2		15.2		7.2		6.9		5.0
50 < 60		14.3		10.7		11.7		6.3		14.5		10.9
60 < 70		11.8		10.1		10.8		12.6		22.3		18.8
70 < 80		10.3		10.8		7.7		22.1		29.6		32.5
≥ 80		6.3		7.5		4.3		26.4		23.1		30.5
Neighborhood income*a*												
1 (lowest)		22.7		27.0		27.3		30.9		28.2		29.7
2		18.6		19.3		20.4		21.4		20.4		20.4
3		18.8		18.8		18.1		17.1		17.9		17.4
4		18.4		16.5		15.8		16.7		15.9		16.1
5 (highest)		18.1		16.2		15.9		10.6		15.3		14.2
Unknown		2.3		2.3		2.5		3.4		2.3		2.2
Previous respiratory visits*b*												
0		68.3		34.2		22.7		31.6		70.4		70.7
1–2		20.5		25.0		22.9		15.3		17.6		18.3
3–5		7.2		17.4		20.4		15.6		6.9		5.6
≥ 6		4.0		23.4		34.0		37.5		5.1		5.2
Previous cardiovascular visits*b*												
0		78.6		76.2		79.6		56.6		19.1		29.2
1–2		8.9		10.2		9.7		12.6		16.9		14.2
3–5		6.0		5.7		4.7		10.6		21.5		14.5
≥ 6		6.5		7.9		6.0		20.3		42.5		42.1
**a**Income quintile of the area of residence, based on data from the 2001 Canada-wide census; residential areas typically include 300–700 people. **b**Number of physician visits between 1 July 2002 and 30 June 2003 for respiratory and cardiovascular ICD-9 codes.												

*Exposure summary.* Measured 24-hr total PM_10_ concentrations ranged from 5.1 to 248.4 μg/m^3^ between the six TEOMs. The mean (± SD) estimate within the population over the entire study period was 29.4 ± 30.7 μg/m^3^, with an interquartile range of 14.1–31.0 μg/m^3^. Modeled smoke-related PM_10_ concentrations from CALPUFF ranged from 0 to 2,497 μg/m^3^, with a population mean (± SD) of 11.4 ± 61.1 μg/m^3^ and an interquartile range of 0–3.5 μg/m^3^. Most exposure cells had values close to zero (i.e., no smoke) on most days, but we estimated very high values when exposure cells were very near active fires. The population-weighted correlation between TEOM and CALPUFF PM_10_ estimates was 0.44 (we took TEOM measurements at six locations, whereas we derived CALPUFF estimates for 2,538 exposure cells). The 92-day correlation between TEOM and CALPUFF PM_10_ estimates at the six TEOM locations ranged from 0.37 to 0.76 (Henderson et al. 2008). Days of SMOKE coverage ranged from 1 to 24 (out of 92) within the cohort, with a mean (± SD) of 13.5 ± 2.4. On days of SMOKE coverage, the population mean PM_10_ values for TEOM and CALPUFF were 45.9 ± 42.3 and 44.2 ± 128.8 μg/m^3^, respectively, with a correlation of 0.58. On days with no SMOKE coverage, they were 23.1 ± 20.8 and 4.3 ± 31.3 μg/m^3^, respectively, with a correlation of 0.31.

*General health outcomes.* Respiratory physician visits were positively associated with all exposure metrics, but the estimate [OR = 1.05; 95% confidence interval (CI), 1.03–1.06] was significant (*p* < 0.05) only for a 30-μg/m^3^ increase in total PM_10_ based on TEOM measurements (Table 2). Similarly, we observed an increase in respiratory hospital admissions across all exposure metrics, but the results were significant only for a 30-μg/m^3^ increase in total PM_10_ based on TEOM (OR = 1.15; 95% CI, 1.00–1.29) and a 60-μg/m^3^ increase in smoke-related PM_10_ based on CALPUFF (OR = 1.11; 95% CI, 1.04–1.18) metrics. Results for cardiovascular physician visits and hospital admissions were null. When stratified by age category, the largest ORs for respiratory physician visits were observed in the 30- to 40-year age category (Figure 2A). For cardiovascular visits, we observed significant positive associations only for total PM_10_ (TEOM) and smoke-related PM_10_ (CALPUFF) in the 40- to 50-year age group and for total PM_10_ in the ≥ 80-year-old group (Figure 2B). We found a negative association with SMOKE in the 70- to 80-year age category. We observed no clear differences by sex, socioeconomic status, or possible preexisting sensitivity (data not shown).

**Table 2:** Overall results for each exposure metric (lag 0 in all cases) [OR (95% CI)].

TEOM			CALPUFF (per 60 μg/m^3^)
Outcome	ICD-9	Per 10 μg/m^3^		Per 30 μg/m^3^	SMOKE (1 vs. 0)
Physician visits		All respiratory		1.02 (1.01–1.03)		1.05 (1.03–1.06)		1.01 (0.99–1.03)		1.08 (0.99–1.18)
		Asthma		1.06 (1.03–1.08)		1.16 (1.09–1.23)		1.04 (1.02–1.06)		1.21 (1.00–1.47)
		Cardiovascular		1.00 (0.99–1.01)		1.01 (0.99–1.02)		1.00 (0.98–1.02)		0.98 (0.92–1.05)
Hospital admissions		All respiratory		1.05 (1.00–1.10)		1.15 (1.00–1.29)		1.11 (1.04–1.18)		1.60 (0.90–2.81)
		Cardiovascular		1.00 (0.96–1.05)		1.00 (0.92–1.11)		0.80 (0.60–1.14)		1.12 (0.89–1.66)
All models are adjusted for same-day mean temperature, day of week, and week of study.										

**Figure 3:**
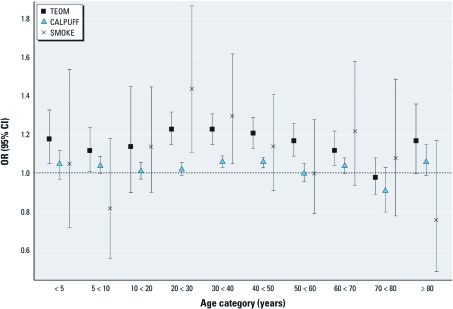
Models for asthma-specific physician visits. All results are for lag 0, and all models are adjusted for same-day mean temperature, day of week, and week of study. The ORs reflect a 30‑μg/m^3^ increase in total (TEOM-measured) PM_10_, a 60‑μg/m^3^ increase in smoke-related (CALPUFF-estimated) PM_10_, and the comparison of SMOKE and no SMOKE.

**Figure 4:**
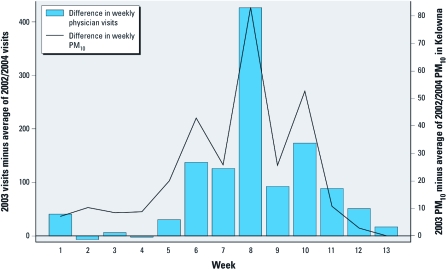
Difference in weekly asthma visits for 2003 versus the average of 2002 and 2004 (when there were few fires in the study area) plotted against the difference in average weekly total PM_10_ measurements for Kelowna, the largest city in the study area. Bars indicate the weekly sum of 2003 asthma-specific visits minus the averages of the 2002 and 2004 weekly sums of asthma visits. The black line indicates the average weekly TEOM-measured PM_10_ in 2003 minus the average of weekly measurements in 2002 and 2004.

*Specific diagnoses.* The overall asthma-specific OR was 1.16 (95% CI, 1.09–1.23) for a 30-μg/m^3^ increase in TEOM-measured PM_10_, 1.04 (95% CI, 1.02–1.06) for a 60-μg/m^3^ increase in CALPUFF-estimated PM_10_, and 1.21 (95% CI, 1.00–1.47) for the presence versus absence of SMOKE coverage. Asthma visits increased in association with a 30-μg/m^3^ increase in total PM_10_ (TEOM metric) across most age categories (Figure 3). A plot of the difference in weekly asthma visits for 2003 versus 2002 and 2004 (when few fires were in the study area) against the difference in average weekly total PM_10_ estimates for Kelowna (the largest city in the study area) highlights the temporal relation between PM_10_ exposures and asthma visits (Figure 4), as well as the increase in total PM_10_ in conjunction with the increased fire activity in 2003.

Of the 34,771 general respiratory physician visits, approximately 21% were coded as “acute bronchitis” or “acute upper respiratory infection” (ICD-9 codes 465 and 466, respectively). A 30-μg/m^3^ increase in total PM_10_ was not associated with these cases (TEOM OR = 0.93; 95% CI, 0.79–1.10). Similarly, approximately 50% of the 46,037 general cardiovascular visits were coded as “essential hypertension” (ICD-9 code 401), and total PM_10_ was not associated with these cases (TEOM OR = 0.97; 95% CI, 0.88–1.09). When we repeated analyses for cardiovascular physician visits excluding this and another diagnoses for acute rheumatic fever, chronic rheumatic heart disease, and hypertensive disease (ICD-9 codes 390–409), associations with all three exposure metrics remained null.

## Discussion

Overall, we found that a 10-μg/m^3^ increase in total PM_10_ (TEOM) was associated with a 5% increase in the odds of a respiratory hospital admission (OR = 1.05; 95% CI, 1.00–1.10) but not with the odds of a cardiovascular admission (OR = 1.00; 95% CI, 0.96–1.05). In the most similar study published to date, Delfino et al. (2009) reported that a 10-μg/m^3^ increase in PM_2.5_ (PM with diameter ≤ 2.5 µm) was associated with a 3% increase in the relative rate (RR) of respiratory hospital admissions in Los Angeles (RR = 1.03; 95% CI, 1.01–1.04) but not with cardiovascular admissions (RR = 1.01; 95% CI, 0.99–1.02). In the present study, a 10-μg/m^3^ increase in PM_10_ was associated with a 6% increase in the odds of an asthma-specific physician visit (OR = 1.06; 95% CI, 1.03–1.08). In the study by Delfino et al. (2009), a 10-μg/m^3^ increase in PM_2.5_ was associated with a 5% increase in the RR of asthma hospital admissions (OR = 1.05; 95% CI, 1.02–1.08). Although we estimated associations with PM_10_ and Delfino et al. (2009) evaluated PM_2.5_, previous work showed that most PM_10_ in our study area during the fire season consisted of PM_2.5_ (Moore et al. 2006).

Other studies have reported that children (Delfino et al. 2009) and the elderly (Delfino et al. 2009; Kunii et al. 2002) were at higher risk of the respiratory impacts associated with smoke exposure, but our results suggest that associations with total PM_10_ during the forest fire season were strongest in adults. Differences among studies may result from differences in study power, study populations, and/or study designs. To the best of our knowledge, the present work is the first cohort study of forest fire smoke epidemiology. The principal difference between this and other studies is that each member of the cohort contributed exposure profiles for all person-days to the analyses, regardless of their event status. Other studies count only persons experiencing an event. In this cohort, 9.3% of the population comprised children < 10 years of age (Table 1), but they accounted for 12.9% of asthma physician visits, 14.6% of all respiratory physician visits, and 13.9% of respiratory hospital admissions. Similarly, 16.6% of the population was > 70 years old, and they accounted for 48.5% of respiratory hospital admission. As such, study designs that count only events are, by definition, disproportionately weighted toward those subpopulations. The cohort for this study better reflects the general population of the study area, although it comprises individuals who regularly use the health care system and live mainly in urban and suburban areas. Overall, our findings with regard to total PM_10_ and respiratory and cardiovascular outcomes were similar to those reported elsewhere.

We assumed that steep elevations in TEOM-measured PM_10_ were caused by forest fire smoke and observed that weekly average concentrations during the summer of 2003 were higher than those in 2002 and 2004 (Figure 4). Although forest fire smoke was the dominant source of PM in the study area during the study period, the TEOM metric reflects PM_10_ from all sources. The CALPUFF and SMOKE metrics reflect PM from smoke only and are more spatially resolved than the TEOM estimates, but they also have limitations. For example, the CALPUFF model performs poorly under low wind conditions (Henderson et al. 2008), when fires are typically smoldering, and PM concentrations are high. Because the plume trajectories tend to be inaccurate under such conditions, PM is underestimated in areas that are actually exposed. This is consistent with our respiratory results, with Table 1 and Figures 2A and 3 showing that the ORs for CALPUFF generally track those for TEOM (same direction in 16 of 22 cases) but are attenuated toward the null. For the SMOKE metric, we might expect less spatial misclassification of exposure, but cells covered by a smoke plume in only one of several satellite overpasses on a single day were treated the same as cells covered in multiple overpasses on the same day. The ORs for the presence or absence of SMOKE tracked slightly less well with ORs for total PM_10_ based on TEOM (same direction in 14 of 22 cases), having larger effect estimates in many cases, with wider CIs (not surprising given the binary nature of the variable). Although the agreement with results based on PM_10_ estimates was limited, it suggests that purely satellite-derived estimates of smoke exposure may be useful after further refinement.

Despite epidemiologic evidence suggesting mainly respiratory effects, human toxicologic studies indicate that biomass smoke is associated with acute outcomes that pose some cardiovascular health risk (Barregard et al. 2006; Swiston et al. 2008; Tan et al. 2000). However, other work suggests that the inflammatory potential of wood smoke particles is less than that of urban particles for cells exposed for > 12 hr (Kocbacha et al. 2008) and that biomass smoke particles deposit less efficiently than do urban (traffic-related) particles in the human respiratory tract (Londahl et al. 2008, 2009). Given the weight of evidence suggesting a relationship between PM exposure and cardiovascular health (Naeher et al. 2007), the impact of fire smoke on cardiovascular health still requires further investigation.

Likewise, effects of chronic exposure to wildfire smoke have yet to be studied but could pose a considerable health risk in areas where annual burning is practiced. In the cases of both acute and chronic exposure, further exploration and refinement of metrics based on remote-sensing data may produce quick, accurate, and spatially resolved exposure metrics, especially in heavily affected areas where air quality monitoring is practically nonexistent. Although we have also demonstrated that dispersion modeling of exposure can produce epidemiologic results similar to those from air quality measurements, the data and resources necessary to run dispersion models may not be available in the aforementioned areas.

## Supplemental Material

(420 KB) PDF
