# Supplementary material for: Three Measures of Forest Fire Smoke Exposure and Their Associations with Respiratory and Cardiovascular Health Outcomes in a Population-Based Cohort
Source: Environ Health Perspect. 2011 Jun 9;119(9):1266–71. doi: 10.1289/ehp.1002288 (PMC3230386; doi:10.1289/ehp.1002288)
Supplement: (420 KB) PDF [file ehp.1002288.s001.pdf]

Supplemental Material

Three Measures of Forest Fire Smoke Exposure and Their Associations with Respiratory and Cardiovascular Health Outcomes in a Population-Based Cohort

Sarah B. Henderson[1*]
Michael Brauer[1]
Ying C. MacNab[2]
Susan M. Kennedy[1,2]

[1]School of Environmental Health, The University of British Columbia, Vancouver, BC, Canada

[2]School of Population and Public Health, The University of British Columbia, Vancouver, BC, Canada

[*]Sarah B. Henderson
School of Environmental Health
The University of British Columbia
3rd Floor, 2206 East Mall
Vancouver, BC V6T 1Z3, Canada
Phone: 604-822-1274
Fax: 604-822-9588
Email: sarah.henderson@ubc.ca

Table of Figures

Supplemental Material, Figure 1 3
Supplemental Material, Figure 2 4
Supplemental Material, Figure 3 4
Supplemental Material, Figure 4 5
Supplemental Material, Figure 5 5
Supplemental Material, Figure 6 6

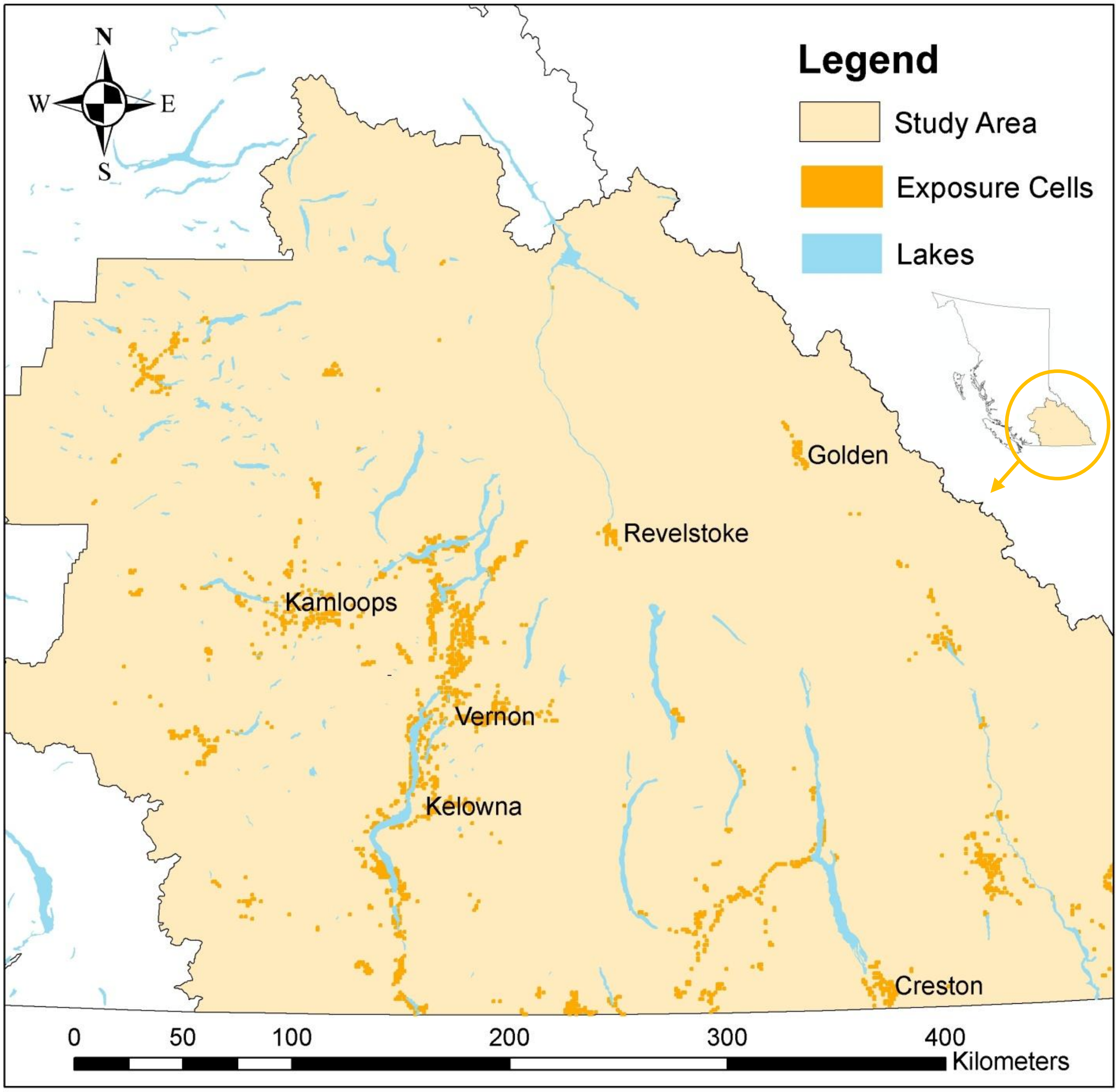

Supplemental Material, Figure 1

The study area. Labelled cities indicate the location of regulatory PM_{10} (TEOM) monitors. Darker orange areas show the location of exposure cells as described in the Methods section of the main text.

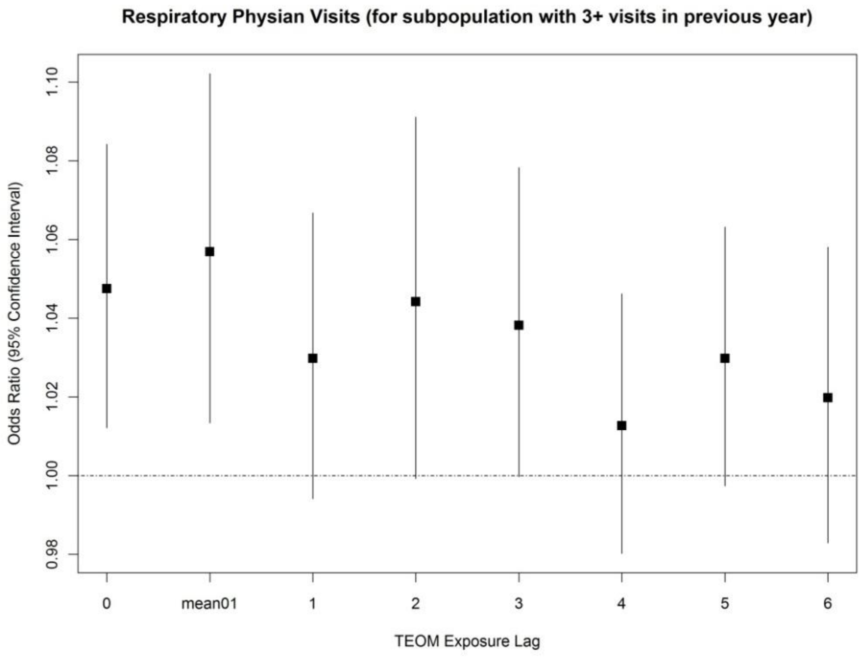

Supplemental Material, Figure 2

Lagged effect of TEOM PM_{10} on all respiratory physician visits for the subpopulation of people with 3+ visits in the year prior to the study period (subsequent analyses found no effect modification by number of previous visits). Lag 0 is reported in the main manuscript for all exposure metrics.

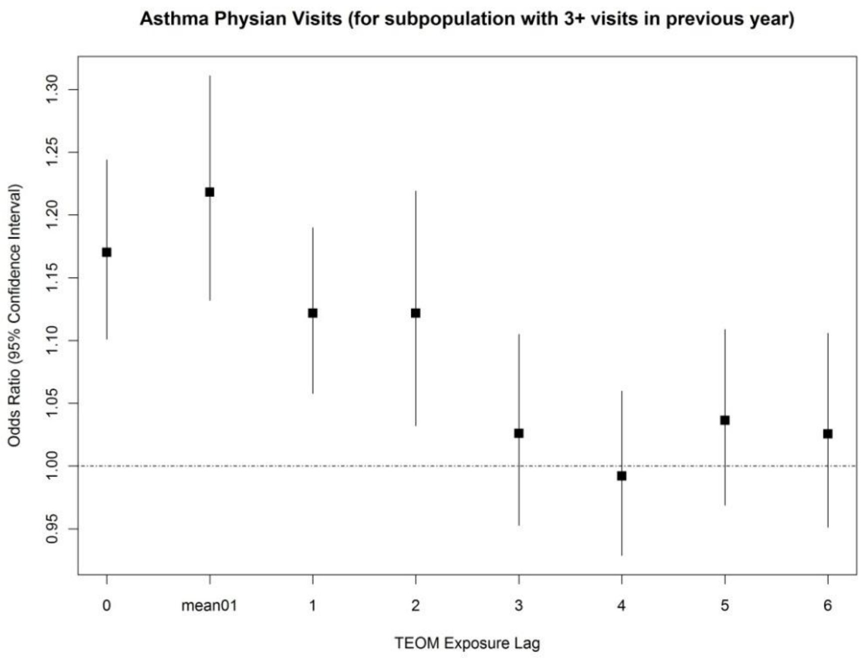

Supplemental Material, Figure 3

Lagged effect of TEOM PM_{10} on asthma-specific physician visits for the subpopulation of people with 3+ visits in the year prior to the study period (subsequent analyses found no effect modification by number of previous visits). Lag 0 is reported in the main manuscript for all exposure metrics.

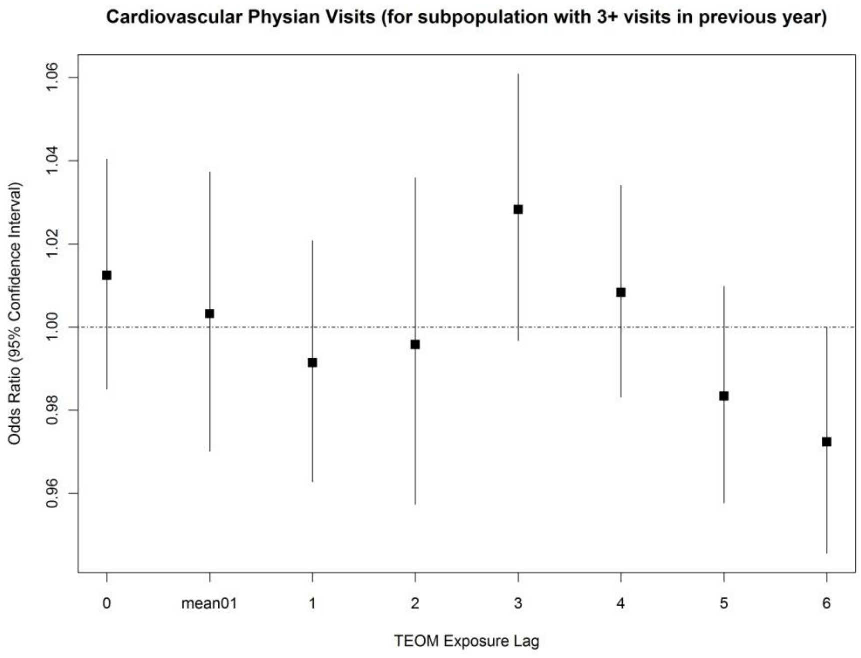

Supplemental Material, Figure 4

Lagged effect of TEOM PM_{10} on cardiovascular physician visits for the subpopulation of people with 3+ visits in the year prior to the study period (subsequent analyses found no effect modification by number of previous visits). Lag 0 is reported in the main manuscript for all exposure metrics.

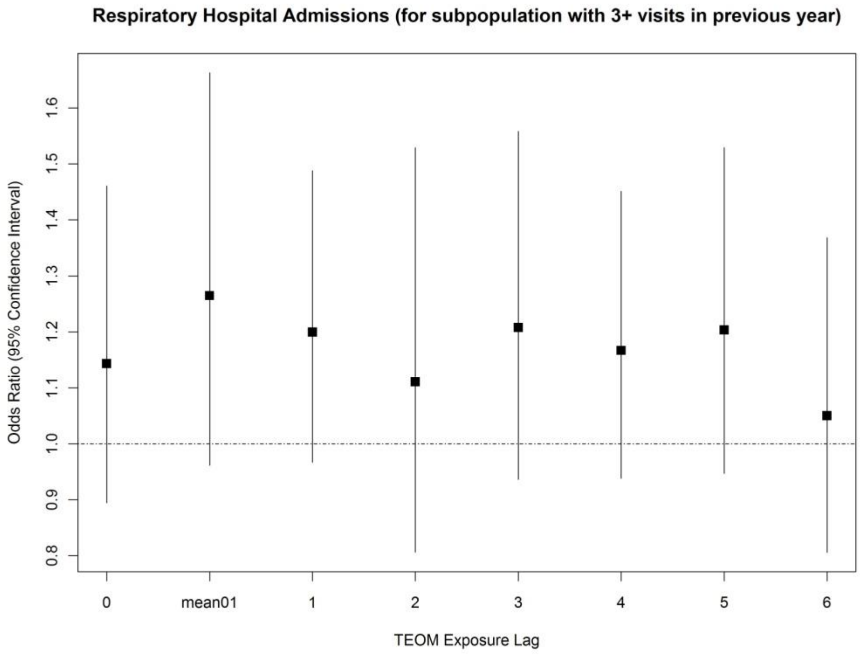

Supplemental Material, Figure 5

Lagged effect of TEOM PM_{10} on respiratory hospital admissions for the subpopulation of people with 3+ visits in the year prior to the study period (subsequent analyses found no effect modification by number of previous visits). Lag 0 is reported in the main manuscript for all exposure metrics.

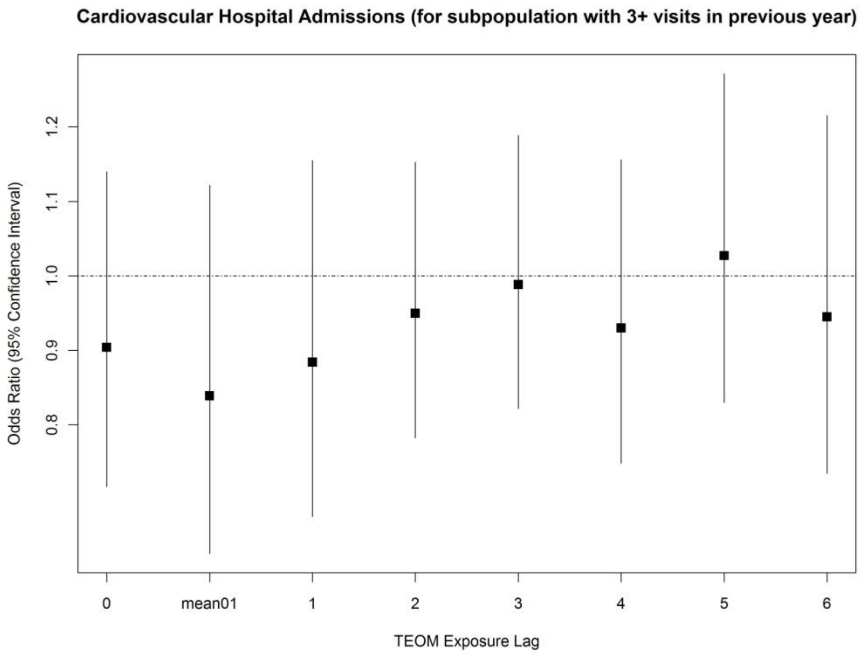

Supplemental Material, Figure 6

Lagged effect of TEOM PM_{10} on cardiovascular hospital admissions for the subpopulation of people with 3+ visits in the year prior to the study period (subsequent analyses found no effect modification by number of previous visits). Lag 0 is reported in the main manuscript for all exposure metrics.
